# Relapse of pathological angiogenesis: functional role of the basement membrane and potential treatment strategies

**DOI:** 10.1038/s12276-021-00566-2

**Published:** 2021-02-15

**Authors:** Anthony Mukwaya, Lasse Jensen, Neil Lagali

**Affiliations:** 1 Division of Ophthalmology, Department of Biomedical and Clinical Sciences, Faculty of Medicine, Linköping University, Linköping, Sweden; 2 Division of Cardiovascular Medicine, Department of Health, Medicine and Caring Sciences, Faculty of Medicine, Linköping University, Linköping, Sweden; 3 Department of Ophthalmology, Sørlandet Hospital Arendal, Arendal, Norway

**Keywords:** Inflammation, Experimental models of disease

## Abstract

Blinding eye diseases such as corneal neovascularization, proliferative diabetic retinopathy, and age-related macular degeneration are driven by pathological angiogenesis. In cancer, angiogenesis is key for tumor growth and metastasis. Current antiangiogenic treatments applied clinically interfere with the VEGF signaling pathway—the main angiogenic pathway—to inhibit angiogenesis. These treatments are, however, only partially effective in regressing new pathologic vessels, and the disease relapses following cessation of treatment. Moreover, the relapse of pathological angiogenesis can be rapid, aggressive and more difficult to treat than angiogenesis in the initial phase. The manner in which relapse occurs is poorly understood; however, recent studies have begun to shed light on the mechanisms underlying the revascularization process. Hypotheses have been generated to explain the rapid angiogenic relapse and increased resistance of relapsed disease to treatment. In this context, the present review summarizes knowledge of the various mechanisms of disease relapse gained from different experimental models of pathological angiogenesis. In addition, the basement membrane—a remnant of regressed vessels—is examined in detail to discuss its potential role in disease relapse. Finally, approaches for gaining a better understanding of the relapse process are discussed, including prospects for the management of relapse in the context of disease.

## Blood vessel formation: understanding relapse after inhibitory treatment

Studying the sites of previous blood vessel occupation may explain how pathological vessel growth recurs after inhibitory treatment. Angiogenesis, the growth of new blood vessels, drives progression of cancerous tumors and blinding eye diseases. Existing treatments inhibiting critical growth factors are effective in the short term, but are often followed by aggressive relapse. Mukwaya et al., from Linköping University in Sweden review the role of empty basement membrane sleeves (EBMS), the remnants of regressed blood vessels. Basement membrane supports endothelial cells, and EBMS could act as a scaffold for endothelial cells to rapidly rebuild blood vessels after anti-angiogenic treatment. Alternatively, EBMS may represent irreversibly abandoned conduits permanently isolated from the circulation, putatively through the deposition of excess collagen. Future work should carefully examine the role of basement membrane in the relapse of pathologic vessel growth.

## Introduction

**Fig. 1:**
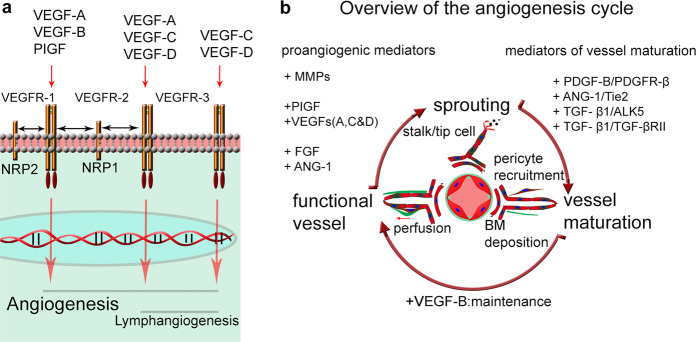
The key signaling molecules in angiogenesis. **a** Simplified schematic illustration of the VEGF family ligands VEGF-A, -B, -C, and -D interacting with their receptors VEGR-1, -2 and -3 to drive lymphangiogenesis and angiogenesis. Neuropilin receptors (NRP1 and NRP2) enhance VEGF signaling by acting as coreceptors for VEGFR-1 and VEGFR-2. **b** Angiogenesis, the growth of new blood vessels from pre-existing vessels, is regulated mainly by the VEGF signaling pathway. Growth factors in the VEGF family, such as VEGF-A, VEGF-C, VEGF-D, and PlGF, as well as other factors, such as the fibroblast growth factor (FGF) family, angiopoietin-1 (Ang-1), and MMPs, are important for the sprouting of new vessels. The newly formed blood vessels are initially leaky but over time undergo maturation characterized by deposition of BM pericyte and mural cell coverage, regulated by pathways such as PDGF-B signaling via PDGFR-B and ANG-1 signaling via Tie2, and TGF-β^133^. Mature blood vessels are maintained in a functional state by factors such as VEGF-B. Under pathological conditions, such as in the tumor microenvironment, these processes are dysregulated, leading to poorly developed vessels that leak fluid and form a disorganized vascular network^134^.

Angiogenesis is the growth of new blood vessels from a pre-existing vascular plexus and is an important process characterizing the progression of pathological conditions such as tumors^1^ and blinding eye diseases such as age-related macular degeneration (AMD), diabetic retinopathy^2^, and corneal neovascularization^3^. The angiogenic process involves the breakdown of endothelial cell (EC) junctions, for example, mediated by vascular endothelial growth factor (VEGF)-induced VE–cadherin endocytosis^4^. This process leads to vessel dilation, degradation of the basement membrane (BM), and EC proliferation in response to this angiogenic growth factor. BM degradation is mediated by the proteolytic activity of matrix metalloproteinases (MMPs), such as MMP-2 and MMP-9, which are largely recognized as key players in angiogenesis^5,6^. ECs in their quiescent state do not express active MMPs; however, under certain conditions, such as during inflammation, a common denominator in pathological angiogenesis, ECs are activated to express abundant levels of these proteases^7^. The activity of MMPs is regulated endogenously by tissue inhibitors of metalloproteinases^8^. The VEGF family of ligands in mammals is composed of secreted related growth factor members, including VEGF-A, VEGF-B, VEGF-C, VEGF-D, and placental growth factor (PlGF). The biological function of these ligands is mediated by protein kinase receptors (VEGFRs), i.e., VEGFR-1, −2, and −3^9^. The ligands interact with receptors with varying affinities. In addition, the ligands interact with the nontyrosine kinase receptors neuropilin-1 and neuropilin-2 (Fig. 1). VEGF-A binds principally to VEGFR-2, a cell surface receptor on vascular ECs, leading to increased permeability of vessels, the expression of mediators of BM and ECM degradation, and the proliferation and migration of ECs, leading to sprouting angiogenesis^10^.

The angiogenic front of the sprouting vessel is characterized by two EC phenotypes: stalk and tip ECs. The acquisition of these cell phenotypes is highly regulated. Upregulation of VEGFR-2 expression promotes tip cell behavior, and within stalk cells, DLL4-Notch signaling is activated to limit VEGFR-2 expression, thus maintaining a proliferative but nonmigratory stalk cell phenotype^11,12^. The VEGF/Notch signaling pathway regulates the selection of tip and stalk cells to facilitate directionality at the angiogenic front^13^. The tip EC migrates through the degraded extracellular matrix (ECM) with the aid of filopodia, following the concentration gradient of the secreted growth factor(s), while the stalk cell lags^14^. The newly formed vessels are initially leaky, immature, and disorganized but become mature over time through the deposition of BM around the growing vessel and by pericyte coverage. The formed vasculature is maintained in a functional state by the expression of genes such as VEGF-B, which has been shown to be important for the maintenance of vessels in the cornea^15^.

Most studies investigating pathological angiogenesis have focused on how to regress pathological vessels, with little attention given to the concept of disease relapse, even though disease relapse is a major clinical challenge in cancers and in blinding eye diseases^16,17^. To date, a few studies have investigated this phenomenon, putting forward different theories to explain rapid disease relapse^17–19^. The current knowledge, however, is based on different theories derived from different experimental models of pathological angiogenesis. There is a need to summarize and carefully analyze this knowledge, as understanding it is an important step for advancing the knowledge of disease relapse through further study and developing strategies for more effective therapeutic outcomes when treating disease relapse. This review summarizes the current knowledge of the described mechanisms for rapid disease relapse in different contexts of pathological angiogenesis. In addition, the role of the basement membrane—a remnant of regressed vessels—is examined in detail in relation to its role in promoting or inhibiting rapid disease relapse.

### Vessel regression may define revascularization

EC apoptosis is one of the key stages in capillary regression and is thought to be triggered by a lack of hemodynamic forces and by macrophages in response to a diminished angiogenic microenvironment^20^. Meeson et al. suggested that EC apoptosis during capillary regression is sequential and can be attributed to two causes^21^. The first cause is described to be macrophage dependent. While studying regression of the pupillary membrane, a transient capillary network located in the anterior chamber of the developing eye, Lang et al. noted that the pupillary membrane consisted of ECs in a network of capillaries and that cell death during capillary regression was caused by apoptosis. They observed that apoptosis occurred either within a single EC in a healthy vessel or synchronously along the entire segment of the capillary segment. They speculated that apoptosis was mediated by macrophages, that elicited cell death of a target EC^22^. In line with this, liposome-mediated macrophage depletion in the eye resulted in the persistence of ECs and vessels, which would have regressed normally in the presence of macrophages. Rescue experiments involving the introduction of bone marrow-derived macrophages induced apoptosis and capillary regression^23^. In another study, macrophages were found to induce the apoptosis of ECs and pericytes in a cell cycle-dependent manner, specifically in the G1 phase^24^.

A second suggested cause of regression by EC death, as proposed by Meeson et al., involves the coordinated apoptosis of vascular endothelial cells mediated by lack of blood flow^21^. ECs sense shear stress (resulting from flow) through adhesive protein activation, glycocalyx elongation, bending of primary cilia, caveolae-mediated regulation of Ca+, ion channel activation, G protein-coupled receptor activation, and tyrosine kinase receptor activation^25,26^. These mechanisms are the means by which ECs adopt a specific cell shape and function. In veins, for instance, ECs are polygonal in shape, while arterial ECs are spindle-like and point in the direction of flow^27^. The magnitude, temporal characteristics and spatial gradient of fluid shear stress are key determinants of the EC transcriptome^28,29^. Previous studies showed that shear stress-aligned ECs exhibited altered junctional inclination^30^, reduced the expression of nitric oxide following reversed flow^31^, and reduced vessel permeability^32^. A study by Wang et al. showed that the direction of flow was an important stimulus for regulating the EC gene expression profile, as activation of the eNOS pathway was maximal when ECs were parallel to the flow, whereas NFkB signaling was active when the ECs were perpendicular to the direction of the flow^33^.

**Fig. 2:**
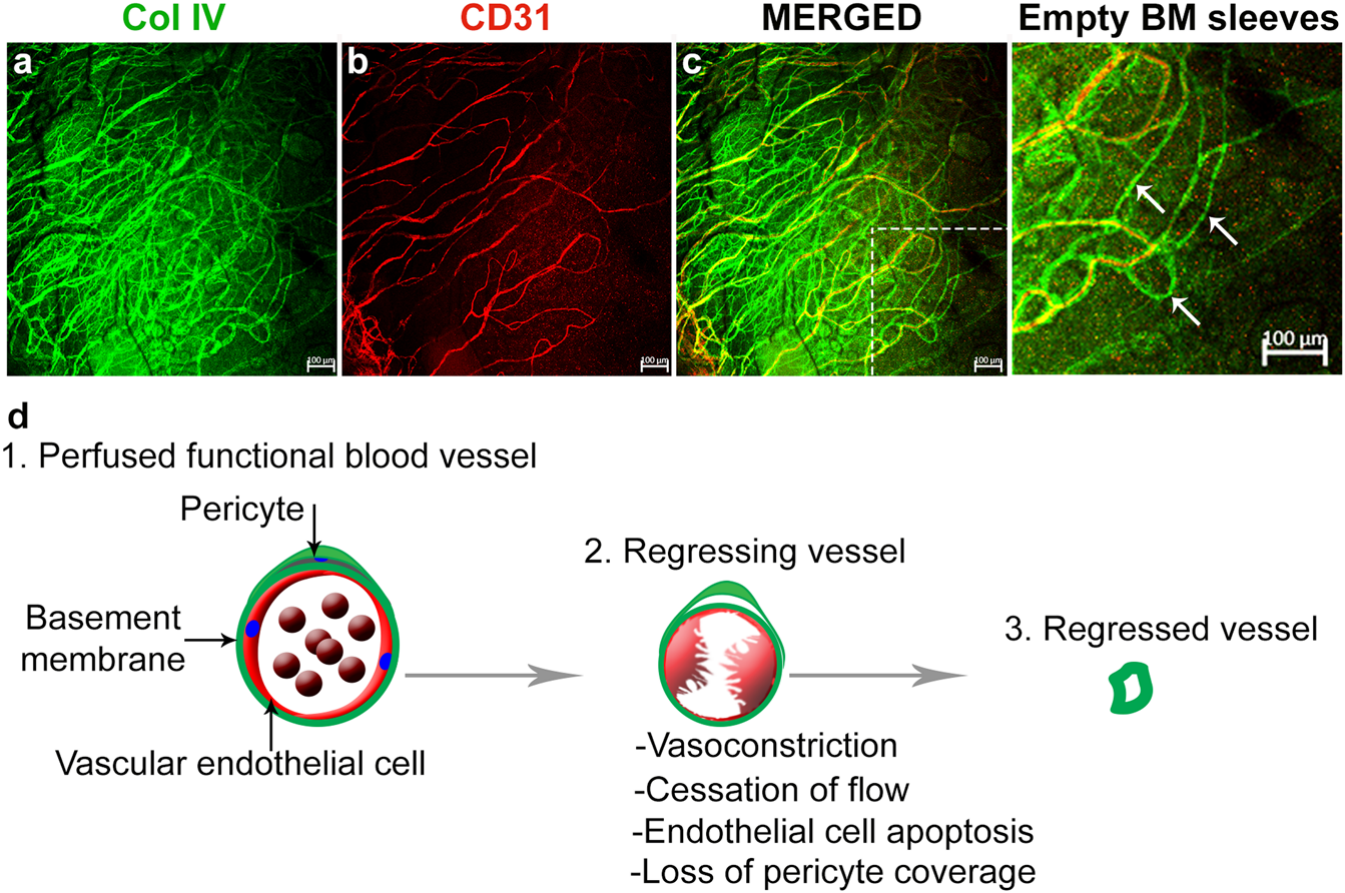
A stepwise loss of the vascular endothelium drives vessel regression. **a**–**c** Regressed blood vessels are devoid of the vascular endothelium (CD31) but retain the basement membrane which is positive for type IV collagen (CoI IV) (arrows) in the magnified region of C as indicated by dashed lines. **d** Schematic representation of the process of capillary regression as observed in a suture induced model of inflammatory corneal angiogenesis. Functional vessels undergo vasoconstriction, cessation of blood flow, apoptosis of the vascular endothelial cells and loss of supporting pericytes, leaving behind empty basement membrane sleeves^18^.

In vitro, laminar shear stress was shown to suppress the expression of the proapoptotic Fas receptor and upregulate the antiapoptotic genes FasExo6Del and Bcl-x(L) in HUVECs^34^. Low shear stress, on the other hand, induces EC apoptosis by activating Akt signaling and producing increased intracellular levels of reactive oxygen species^35,36^. Shear stress may also regulate apoptosis in ECs through cytochrome C release from mitochondria^37^ and trophoblast-induced EC apoptosis^38^ or via PI3K/Akt-dependent signaling^39^. Interestingly, a recent study showed that blocking EC apoptosis in regressing retinal vessels promotes ischemic tissue revascularization. The preserved ECs were found to reassemble into functional networks to restore the blood supply rapidly following exposure to hypoxia^40^. Another study showed that the proinflammatory mediators interleukin-1β (IL), tumor necrosis factor α (TNF), and thrombin regulate vessel regression because of increased levels of phospho-p38 and phospho-MLC2 and decreased levels of phospho-Pak2, acetylated tubulin, phospho-cofilin, and pro-caspase3^41^. In general, regressed vessels are devoid of ECs, and they leave behind empty basement sleeves (embs, i.e., the remnants of regressed vessels), which are imprints of a previous vascular network, as shown here by immunohistochemistry to identify the basement membrane marker type IV collagen and the vascular endothelium marker CD31 (Fig. 2).

### Treatment of pathological angiogenesis

Antiangiogenic therapies targeting growth factors such as VEGF are used to induce regression of pathological vessels^42,43^. Aptamers such as Macugen were developed as antiangiogenic agents that bind human VEGF-A to prevent signaling via VEGF receptors, thus inhibiting activation of a downstream signaling cascade to inhibit pathological angiogenesis^44^. Bevacizumab is a commonly used antiangiogenic agent. Bevacizumab is a recombinant full-length humanized monoclonal antibody against VEGF-A_165_ that binds and blocks the biologically active forms of VEGF to inhibit angiogenesis^45^. Bevacizumab was initially approved for the treatment of colon cancer but is now used as an off-label treatment of various cancers and blinding eye diseases^46^. Ranibizumab is an anti-VEGF agent with the Fab region of the same monoclonal antibody as bevacizumab and is approved for the treatment of neovascular AMD^47^. The efficacy of ranibizumab is similar to that of bevacizumab^48^; however, ranibizumab is more expensive. Aflibercept, also known as VEGF trap, is a decoy receptor for several VEGF family ligands, i.e., VEGF-A, VEGF-B, and PIGF. Aflibercept was created by fusing the immunoglobulin (Ig) domains of VEGFR-1 and VEGFR-2 to the constant region (Fc portion) of human IgG1. Aflibercept binds VEGF-A, VEGF-B, and PlGF with great affinity, thereby inhibiting angiogenesis^49^. Other antiangiogenic treatments include receptor tyrosine kinase inhibitors (TKIs), which compete with ATP for tyrosine kinase-binding sites to reduce tyrosine kinase phosphorylation and thereby inhibit cancer cell proliferation. VEGFR TKIs, such as sorafenib, inhibit RAF-1, and VEGFR-3 activity^50^, and sunitinib, a multitarget kinase inhibitor, targets VEGFR, PDGFR-α, and PDGFR-β^51^. Despite being approved for cancer treatment, rapid emergence of resistance followed by increased tumor invasiveness limits the clinical benefit of sunitinib as an angiogenesis inhibitor^52,53^, and similarly, innate and acquired resistance to sorafenib is common^54^. Treatment of pathological angiogenesis remains a major clinical challenge regardless of the drug used. Targeting a specific growth factor (such as VEGF) can lead to toxicity, nonresponse among patients, and resistance of the pathological vessels to the modulation of a specific pathway^55,56^. Furthermore, relapse of the disease is reported following cessation of treatment in both cancer and eye diseases; however, how relapse occurs is not fully understood.

## Relapse of pathological angiogenesis

In many cancers, anti-VEGF treatment must be given continuously, but this leads to toxicity, worsening the quality of life and/or exacerbating drug resistance mediated by treatment-induced epigenetic changes; therefore, a treatment ‘holiday’ may be required to allow reversion to a previous epigenetic profile^57^. However, relapse of the pathology can occur during these breaks from treatment^58,59^. In the eye, AMD is a chronic and progressive disease affecting the central retina. AMD causes blindness mainly among the elderly population in the developed world^60^ and is classified as either nonexudative, also known as dry AMD; exudative, also known as wet AMD; or neovascular (nAMD)^61^. nAMD is characterized by an ingrowth of pathologic blood vessels from the choroid into the macular region of the retina^62^ and is the cause of complications such as subretinal hemorrhage, vitreous hemorrhage, fibrosis, and scarring, which lead to vision loss^63^. nAMD is less frequent than dry AMD; however, nAMD is responsible for the greatest loss of central vision (90%) associated with AMD^64^. Intravitreal injection of anti-VEGF drugs such as ranibizumab, aflibercept or bevacizumab has become the standard first-line therapy for the management of nAMD^16^. Anti-VEGF treatments require 3 monthly doses followed by individualized treatment (called Treat and Extend), 7–9 injections/year, fixed dosing over 4–8 weeks, or monthly assessment for lesion activity and treatment *pro re nata* (PRN—as needed dosing). These repeated treatments are required because as the effect of anti-VEGF molecules subsides, the disease relapses. Repeated therapy places a large economic burden on health care systems^65,66^, and these treatments maintain but do not improve overall vision. Moreover, anti-VEGF therapies may be associated with adverse side effects such as increased intraocular pressure^67^, and there is a growing concern of acquired resistance to anti-VEGF^56,68^. There is also a group with innate resistance, so-called anti-VEGF “nonresponders”, in which vision loss is characterized by subretinal hemorrhage with persistent fluid/blood leakage^47^. Cessation of regular anti-VEGF treatment, instituted due to side effects or for other reasons, carries a risk of relapse of choroidal neovascularization in nAMD. Numerous studies have reported relapse of pathological angiogenesis following treatment stoppage. For instance, there is a high risk for relapse of retinopathy of prematurity following monotherapy with intravitreal injection of ranibizumab^69,70^ and after intravitreal bevacizumab monotherapy^71^. Following a long-term follow-up, myopic choroidal neovascularization was observed to recur in approximately 40% of patients after intravitreal anti-vascular treatment^72^. Other study estimates show approximately 30% recurrence of choroidal neovascularization in patients treated with anti-VEGF^16,73^, and despite these reports, little is understood regarding how recurrence occurs.

At the front of the eye, corneal neovascularization is the ingrowth of pathological vessels from the peripheral limbus toward the center of the normally clear and avascular cornea. A vascularized cornea, however, impairs vision by absorbing and scattering light and triggering a fibrotic wound healing response that irreversibly alters the normally transparent collagen structure of the cornea. Estimates indicate that corneal neovascularization affects 1.4 million people annually, with 12% of these people losing their vision^74^. Currently, there are no approved treatments for corneal neovascularization; however, VEGF is widely targeted to treating corneal neovascularization. Bevacizumab, for example, has been used off-label for treating corneal neovascularization. In clinical and experimental settings, recurrence of corneal neovascularization has been noted^75,76^. Recurrence of corneal neovascularization as high as 35% was shown in a prospective interventional case series study following the discontinuation of subconjunctival injections of bevacizumab^77^. Dexamethasone—a corticosteroid—is effective in regressing pathological vessels in the cornea^78^; however, this treatment was found to be ineffective for regressing recurrent vessels in a cornea model of pathological angiogenesis^79^.

In the field of cancer therapy, tumor rebound has been documented, for example, in a case report of a patient with recurrent high-grade glioma. Following discontinuation of bevacizumab treatment, tumor regrowth was found to be more aggressive and treatment-resistant^80^. In another case report, tumor rebound was noted after discontinuation of aflibercept treatment in metastatic colorectal carcinoma^81^. Similarly, in preclinical models, the discontinuation of anti-VEGF therapy has been shown to promote metastasis via liver revascularization^59^.

These reports highlight the challenge presented by disease relapse; however, currently there are no treatments specifically designed to address this phenomenon. Furthermore, the process of disease relapse is still poorly understood at the functional level of angiogenic vessels. To better address this situation, it is important to keep in mind how vessels regress (discussed above) and how this regression may contribute to the regrowth of vessels under the appropriate conditions.

### The process of pathological angiogenesis relapse and the role of the basement membrane

**Fig. 3:**
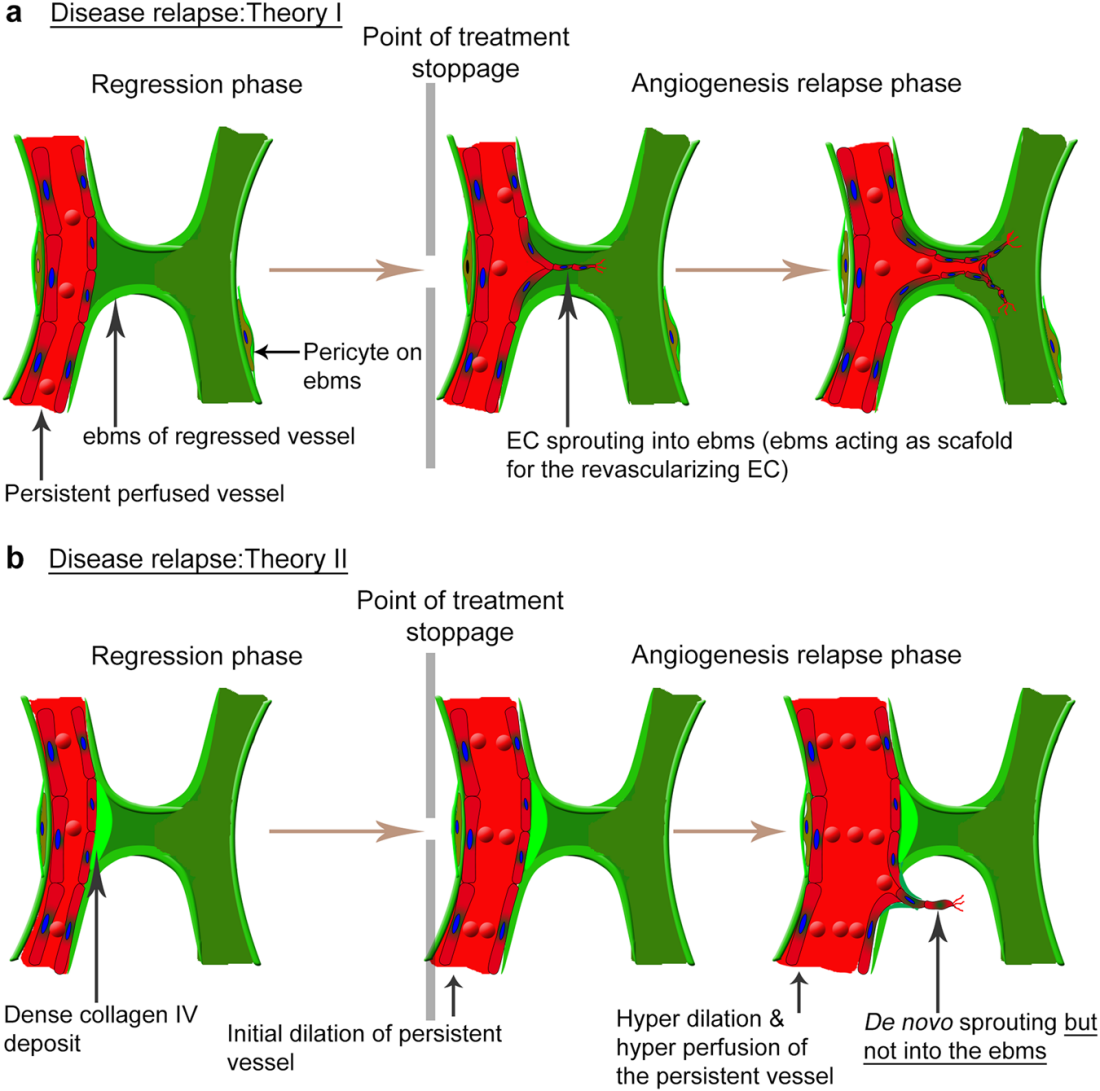
Theories accoutning for the rapid relpase of pathological angiogenesis. **a** Rapid relapse of tumor angiogenesis is hypothesized to be facilitated by the empty basement membrane sleeves (ebms) of previously regressed vessels, which are postulated to serve as scaffolding for ECs during new angiogenic sprouting, following cessation of antiangiogenic treatment^19^. In contrast, **b** rapid relapse of corneal neovascularization is facilitated by dilation and reperfusion of the persistent (treatment-resistant vessels) vessels and by de novo sprouting after vessel reperfusion, which do not involve using the ebms as scaffold^18^.

Little is known about the processes characterizing the rapid recurrence of pathological angiogenesis following cessation of treatment for tumors and ocular angiogenesis. Only a few recent studies have examined the relapse of pathological angiogenesis in great detail. Using the RIP-Tag2 model of tumor angiogenesis in mice, it was shown that pathological vessels regressed following anti-VEGF treatment; however, relapse occurred by EC reoccupation of the ebms once treatment was stopped^19^. The ebms were postulated to serve as a scaffold for revascularizing ECs, thus facilitating rapid revascularization, as illustrated in Fig. 3.

The study, however, only examined a short-term (7-day) inhibition of pathological angiogenesis, which does not mimic a typical clinical treatment strategy, where treatment typically lasts for several weeks or months. It is of interest to know if a similar rate and pattern of disease relapse are observable after long-term VEGF inhibition. With sustained anti-VEGF treatment over a long period, ebms may not persist long enough to facilitate disease relapse. These aspects are important to address to gain a better understanding of the dynamics of tumor relapse under different treatment time frames. However, despite the lack of detailed knowledge concerning these aspects, the repopulation of initially abandoned ebms is accepted as the de facto mechanism of tumor angiogenic relapse following cessation of treatment.

Tumors treated with combretastatin A4 phosphate, a compound that selectively disrupts blood vessels, were found to be rapidly repopulated with TIE2-expressing macrophages (TEMs), leading to increased expression of the chemokine CXCL12. Depletion of TEMs improved the efficacy of combretastatin A4 phosphate in inhibiting angiogenesis^82^. This finding illustrates that macrophages promote the rapid relapse of tumor angiogenesis by facilitating a proangiogenic environment by expressing chemokines important for cell migration. Therefore, determining whether TEMs are critical for the relapse of tumor growth following anti-VEGF treatment in various types of cancer is an interesting study objective.

The term tumor microenvironment is often used to describe the tumor in its entirety, including the vascular network, connective tissue, infiltrating immune cells, and ECM. The ECM constitutes up to 60% of the tumor mass, and it differs greatly from that of normal organs. Processes key for tumor survival, such as intertumoral signaling, transport mechanisms, oxygenation, and metabolism, are all potentially regulated by the ECM. Several studies reviewed by Henke et al.^83^ show that the ECM is important for the outcomes of various forms of cancer therapy, for example, by acting as a barrier protecting tumor cells from the actions of a therapeutic agent.

The extracellular matrix (ECM) is a complex network of polysaccharides, proteoglycans, glycoproteins, and proteins and is a key determinant of the physical and mechanical properties of the tissue, with profound effects on cell behavior^84^. In terms of angiogenesis, studies have shown that the interaction between ECM components and EC receptors mediates EC growth, survival and differentiation^85^. The different components of the ECM, such as fibronectin and thrombospondins, are discussed in greater detail in later sections.

We recently investigated in detail the process of relapse of corneal neovascularization using a rat model of suture-induced inflammatory angiogenesis^86,87^. In this model, a month-long period of vascular regression was induced followed by relapse. The relapse of neovascularization was rapid and more aggressive compared to the initial phase of angiogenesis^79^, corroborating observations in tumor studies. Initially, the examination of the regressed corneal vascular bed indicated presence of many ebms with a few persisting blood vessels. Examination of the persistent blood vessels by electron microscopy indicated the presence of a degenerating vascular endothelium, basement membrane and supporting pericytes. During a secondary stimulus to induce relapse of corneal neovascularization, the degenerating blood vessels rapidly normalized via EC recovery, vasodilation, and perfusion with tightly packed erythrocytes, facilitating the relapse of corneal neovascularization. In this model, ebms did not serve as conduits for revascularizing ECs^18^. Rather, during the vascular regression period, the vessels were devoid of ECs, leaving behind ebms. Excess basement membrane probably expressed by the ECs, was deposited at junctional points of the ebms with few remaining nonregressed functional vessels to irreversibly “seal off” the ebms from blood flow, thus preventing sprouting into these structures during revascularization^18^. In the RIP-Tag2 tumor model from which revascularization hypothesis II was generated, a similar basement membrane deposition pattern was noticeable; however, this excess basement membrane deposition was interpreted to represent basement membrane heterogeneity^19^. Although observations in the cornea model appear to contradict observations previously reported for the RIP-Tag2 tumor model, it is notably important to note that the two studies were conducted in different models, and importantly, the longer period for vascular regression in the cornea model appeared to allow irreversible changes in the ebms. When recapitulated in the cornea model, a shorter 7-day period of regression led to only partial abandonment of the basement membrane sleeves, which retained some ECs and were not sealed off from the blood flow^18^. These ECs would presumably have the potential, as in the tumor model, to rapidly repopulate the still functional basement membrane sleeves. Therefore, the duration of antiangiogenic treatment, and not necessarily the model or tissue, may play a key role in determining the functionality of basement membrane sleeves in facilitating relapse. In support of the idea of EC recovery for revascularization as observed in corneal revascularization, a recent study showed that preventing EC apoptosis was key for the revascularization of an ischemic retina. It was observed that the preserved ECs reassembled and sprouted to revascularize the tissue following exposure to hypoxia^40^.

During the relapse of corneal neovascularization, several proinflammatory and proangiogenic pathways are more strongly activated than are activated during the initial phase of neovascularization. Some of these pathways include leukocyte extravasation, LPS/IL-1–mediated inhibition of RXR function, ILK signaling, production of nitric oxide and ROS signaling, and innate and adaptive immune responses. Genes such as Il-1β and Cxcl2 were increasingly expressed during relapse than during initial angiogenesis^79^. These findings hint at a potential regulatory basis for the relapse of pathological angiogenesis and a potential memory effect, and some of these pathways can be investigated as targets to inhibit or delay angiogenesis relapse. Investigating whether these pathways and inflammation generally have a similar expression pattern in relapsing tumor angiogenesis is an interesting study direction.

In retinopathy of prematurity, failure of the retina to revascularize was associated with senescence, preceded by oxidative stress in the choroid and in the retinal pigment epithelium. These aspects of the disease were thought to be regulated by the suppressed expression of insulin-like growth factor 1 receptor and by the upregulation of the tumor suppressor p53^88^. Therefore, determining whether, for example, promoting p53 signaling can be a means of suppressing revascularization in other tissues and in other contexts of pathological angiogenesis is of interest.

It is unclear whether ebms play any direct role in the relapse of nAMD. A recent study investigating revascularization in a mouse model of ischemic retinopathy showed that preventing EC apoptosis delays vessel regression; however, when the vessels eventually regress, isolated clusters of ECs are preserved within the avascular zone of the tissue. Following a second wave of hypoxia stimulation, these EC clusters rapidly formed vascular networks to revascularize the tissue independent of VEGF-A^40^. In that study, notably, ebms were not used as conduits for revascularization, and sprouting originated from these EC clusters. This mechanism is likely similar to that observed in the cornea model of pathological angiogenesis, and the nonregressed (resistant or persistent) pathological vessels in nAMD may be critical for the relapse of the pathology by dilating and breaking down endothelial barriers leading to the sprouting of new vessels. Recent advancements in retinal imaging provide a more detailed investigation into the mechanisms driving recurrent nAMD. Numerous studies have, for example, used optical coherence tomography angiography (OCT-A) imaging to investigate the vascular response to antiangiogenic treatment by monitoring blood flow patterns. In one study investigating the early effects of treatment discontinuation in choroidal neovascularization, it was noted that anti-VEGF treatment enhanced closure of terminal vessels and that treatment caused increased flow within the trunk during choroidal neovascularization reactivation^89^. In another study, Alexandra et al., using OCT-A, found that after long-term VEGF inhibition, relapse was characterized by sub-RPE neovascularization^90^. Studies such as these are shedding light on the processes of pathological angiogenesis relapse in the retina, and this knowledge may influence future treatment regimens for pathological angiogenesis in this tissue.

## Why empty basement membrane sleeves may or may not facilitate rapid disease relapse

Ebms have long been thought to be merely dormant structures that remain following regression of vessels; however, these structures are now hypothesized to potentially play roles in the rapid relapse of tumor angiogenesis following cessation of short-duration treatment^19^. After a longer duration of antiangiogenic treatment, however, such as in the corneal neovascularization model, ebms were found not to be important for the rapid relapse of the pathology cessation of treatment^18^. It is therefore of interest to examine the potential mechanisms by which the BM—a component of embs—can either support or hinder rapid relapse of pathologic angiogenesis.

**Fig. 4:**
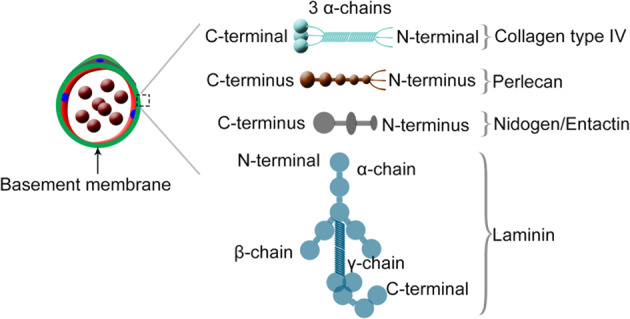
The major structural components of the basement memebrane. Schematic illustration of the four major components of the basement membrane (collagen IV, perlecan, nidogen, and laminin).

The BM is a thin, dense and cross-linked component of the extracellular matrix that borders all cellular monolayers, including the epithelia and endothelia^91^. The BM is synthesized within and secreted by the epithelia or endothelia. BMs were initially identified in muscle and later in nearly all tissues^92^. The BM consists of members of the laminin family, nidogens, heparan sulfate proteoglycans, perlecan, and collagens^91^. Figure 4 shows a schematic of the four major structural components of the BM^93^.

The BM is highly heterogeneous as a result of variations in the relative amounts of the different BM components and the subtypes participating in BM assembly. Variations in type IV collagen and laminin are the main factors influencing tissue-specific BM composition and are thus critical for the functional diversity of the BM. Mechanisms such as splice variants, tissue-specific gene regulation and posttranscriptional modifications define the composition of a specific BM. Proteolytic posttranslational processing of laminins, for instance, modulates their function and binding affinities for other components of the BM, thus influencing overall BM composition^94^.

Under normal physiological conditions, the vascular basement membrane supports the vascular endothelium and acts as a barrier to diapedesis of leukocytes; however, transendothelial migration occurs during embryogenesis or in pathologies such as cancer, during cancer cell metastases^95^, and/or in much more common situations such as inflammation. Migration of cells across the BM is thought to involve several processes, including ICAM-1-mediated leukocyte rolling^96^, local degradation of the BM via expression of matrix metalloproteinases, or cellular invadopodia breaching small imperfections in the BM barrier^97^. Among the many functions of BMs, the ability to sequester and act as a growth factor reservoir is relevant for revascularization. Growth factors such as fibroblast growth factor bind heparan sulfate proteoglycan (HSPG) and are enzymatically cleaved and released as soluble ligands^98^. Cleavage, however, is not believed to be a prerequisite for the functionality of HSPG-bound growth factors, as HS may act as a cofactor presenting ligands during signaling. A previous study analyzing the growth factors in reconstituted basement membrane found that bFGF, EGF, and PDGF were all present in this basement membrane and that, when released, these growth factors promoted cell proliferation^99^. In the bovine cornea, bFGF was found to bind to heparan sulfate, and this binding was speculated to be an inherent mechanism for regulating angiogenesis^100^. This ability of BM to bind and store growth factors may support the theory of its role in facilitating rapid revascularization by acting as scaffold for revascularization by endothelial cells following cessation of antiangiogenic treatment. The rapid rate of the observed revascularization, however, indicate that ECs must divide and migrate very rapidly to repopulate ebms.

### Collagen—a major component of the BM

Collagens constitute a group of proteins characterized by Gly-X-Y motifs, with types I, II, and III being classical examples of collagens containing long Gly-X-Y motifs. The vascular BM consists of varying amounts of type IV, XV, and XVIII collagen. One study found that type XVIII collagen is the predominant collagen type in the BM of capillaries in the lung alveolus. In addition, BM with type XV collagen is predominant in capillaries of the heart, muscle layer of small intestines, and skeletal muscle^101^. Of the collagens, type IV collagen makes up 50% of the entire BM and is essential for BM assembly and stability^102^. Type IV collagen is expressed and secreted as protomers, which are heterodimers composed of three α chains.

### Type XV collagen

Type XV collagen is expressed in various tissues, muscle cells, fibroblasts and ECs and is in the basement membrane of vessels^103,104^. Type XV collagen-deficient mice were shown to develop normal vasculature and vascular BMs^105^. Abnormal capillaries were, however, detected in the heart and skeletal muscle in the bacterium *C. elegans*, which possesses a gene homologous to that coding for Type XV in vertebrates^106^. Cleavage of the C-terminal NCI domain of type XV collagen results in the production of restin, a molecule shown to possess antiangiogenic properties in vitro by inhibiting bFGF-induced EC migration and in a xenograft carcinoma model in vivo^107,108^. Therefore, the degradation of type XV collagen would likely result in the generation of molecules such as restin and endostatin, which would exert antiangiogenic effects during disease relapse.

### Type XVIII collagen

Type XVIII collagen is a precursor of endostatin, a molecule known to possess antiangiogenic properties^109^. Endostatin is a carboxyl-terminal fragment of collagen XVIII generated from protease activity. Similar to angiostatin, endostatin inhibits EC proliferation and was shown to mediate antiangiogenic properties by inhibiting EC proliferation and tumor growth^109,110^. Endostatin is proposed to function by preventing TNF-induced activation of JNK signaling^111^ and by causing cell cycle arrest of ECs^112^. In vitro, endostatin was shown to specifically induce apoptosis of ECs by increasing the activity of caspase 3, leading to DNA degradation. In addition, the expression of the antiapoptotic proteins Bcl2 and Bcl-X_L_ was reduced with endostatin treatment^110^. In another study, endostatin in the presence of bFGF was shown to induce tyrosine kinase phosphorylation of shb adapter protein to enhance the apoptosis rate of ECs^113^. These reports demonstrate the antiangiogenic role of endostatin. If ebms, as remnants of regressed vessels, are digested by proteases during a vascular regression phase, antiangiogenic molecules would be expected to be present in the local ECM during disease relapse, possibly favoring ebms abandonment over their reuse as scaffolding for revascularizing ECs.

### Type IV collagen

Type IV collagen is a major structural component of the BM consisting of six separate α-chains, α1 (IV)–α6 (IV), which assemble into three distinct promoters^114^. During angiogenesis, type IV collagen forms gradually around the developing vascular endothelium, appearing initially in patchy deposits, which later develop into a continuous stabilization structure;^115^ however, the exact regulatory mechanisms underlying this process are not well understood. In one study, type IV collagen was shown in vitro to promote both the proliferation and stabilization of endothelial sprouts in a rat aortic ring assay^116^. The function of type IV collagen in the elongation and stabilization of microcapillaries was dose-dependent; however, with low concentrations of type IV collagen promoting elongation, and high concentrations of type IV collagen stabilizing microcapillaries^116^. In this study, the authors speculated that type IV collagen induced elongation by enhancing morphogenetic recruitment of ECs from the aortic explant, given that previous reports had shown that ECM molecules induced capillary morphogenesis in a planar culture system^117,118^. The role of stabilizing vessels at high concentrations is not limited to type IV collagen, as studies have shown that laminin-entactin also enhances the stability of vessels^119^.

Canstatin is a fragment of the α2 chain of type IV collagen and has been shown to possess antiangiogenic properties. For instance, canstatin reduced the expression of angiopoietin‐1 to inhibit lymphangiogenesis in vitro and in vivo^120^. Similar antiangiogenic and antilymphatic activities have been reported in tumors (reviewed by Mundel et al.)^121,122^. Additionally, Magnon et al. showed that the antiangiogenic properties of canstatin are mediated via mitochondrial damage as a result of integrin-induced caspase-8-promoted apoptosis^123^. A similar mode of action was proposed through the use of a gastric cancer xenograft model^124^. Mechanistically, canstatin inhibits the phosphorylation of key elements involved in the VEGF signaling pathway, such as Akt and focal adhesion kinase, to inhibit cell proliferation and migration^125^. Other described modes of action of canstatin involve the induction of Fas-dependent apoptosis of ECs^126^. Degradation of the BM by proteases results in the generation of canstatin, which mediates angiogenic properties, making the BM less favorable for reuse by revascularizing ECs during disease relapse.

Tumstatin is a 28 kD noncollagenous domain fragment (NC1) cleaved from the α-3 chain of collagen IV and is largely known as an endogenous inhibitor of angiogenesis^127^. A tumstatin peptide was shown to prevent hypertrophy in diabetic nephropathy by inhibiting alterations in glomerular hypertrophy, hyperfiltration and albuminuria^127^. In a streptozotocin-induced diabetes mouse model, tumstatin inhibited inflammatory cell infiltration and the expression of angiogenesis-associated molecules such as VEGF and ANG-2^127^. In another study, tumstatin was shown to bind the collagen-binding domain of MMP2, inhibiting its activation and thus mediating antiangiogenic properties in a laser-induced choroidal neovascularization model^128^. In ECs, tumstatin mediates antiangiogenic properties and promotes apoptosis via the inhibition of Cap-dependent translation (protein synthesis) mediated by the focal adhesion kinase/phosphatidylinositol 3-kinase/Akt/mTOR/4E-BP1 pathway^129^. Ascertaining the exact role of tumstatin during relapse of pathological angiogenesis is an interesting goal, given that this antiangiogenic peptide is produced by MMP-9 proteolysis of the α-3 chain of collagen IV^130^.

Fibronectin, a glycoprotein component of the ECM, is a provisional matrix highly expressed around developing vessels during embryogenesis but is almost undetectable in adult vasculature. The expression of this component of the ECM is, however, found to be reactivated under pathological angiogenesis. A study showed that fibronectin, acting via its receptor integrin α5β1, regulates angiogenic pathways that are distinct from VEGF-mediated angiogenic pathways^83^. In the OIR model where the VEGF receptor tyrosine kinase inhibitor KRN633 was used to block VEGF signaling to establish capillary-free zones in the central and peripheral retinas of neonatal mice, revascularization was found to occur following the cessation of treatment, with the re-formed fibronectin (provisional matrix) serving as a scaffold for revascularization^131^.

Another glycoprotein component of the ECM, thrombospondins (TSP 1 and 2), are known to exhibit antiangiogenic properties. A study showed that TSP1 and TSP2 induce the apoptosis of microvascular ECs by binding to the transmembrane glycoprotein CD36 receptor, impairing EC function and tube formation^132^.

Following the regression of blood vessels, it is not yet clear whether the ebms remain as hollow tubes or whether they collapse soon after EC abandonment. Ascertaining the behavior of these structures over time is of interest because the results will lead to better understanding of the rapid disease relapse process in tumors and corneal neovascularization following the cessation of treatment. It is likely that the reoccupation of collapsed ebms may require activation of a subset of genes different from those required for the reoccupation of hollow ebms. Since embs composed of collagen IV dominate the regressed vascular bed, the facilitation, not necessarily the inhibition of disease relapse, deserves closer investigation given the abundant antiangiogenic properties described for collagen IV and its various protein derivatives, including canstatin, tumstatin, endostatin, and restin.

## Conclusions and future prospects

Pathological angiogenesis is a major clinical challenge in cancer and in eye disease. As the search for more effective therapies for pathological angiogenesis continues, future approaches should aim to inhibit initial angiogenesis more completely to prevent relapse of the pathology. Persistent, incompletely regressed vessels likely serve as the main sources of vessel regrowth and reactivation and ultimately disease relapse. Therefore, a sustained period within an antiangiogenic environment is required to regress active angiogenic vessels and deactivate the remaining ebms. Disease relapse is observed clinically in ocular and tumor angiogenesis and is often managed by a similar regimen as that used to treat the initial angiogenesis. Disease relapse, however, can be more rapid and aggressive than disease in the initial phase. For example, in the cornea, dexamethasone, a corticosteroid shown to efficiently inhibit initial angiogenesis, was found to be ineffective in regressing pathological vessels during disease relapse^79^. This observation may indicate that potentially different regulatory mechanisms drive the process of initial angiogenesis and relapse^79^. Although higher therapeutic doses can be given, the higher levels may increase toxicity and side effects associated with the treatment. Here, two hypotheses were discussed to explain the rapid relapse of pathology after terminating antiangiogenetic treatment. One hypothesis described in the context of tumor angiogenesis implicated ebms as key structures driving rapid revascularization by acting as conduits for EC migration during relapse after a short-duration treatment^19^. Several questions, however, remain unanswered regarding this hypothesis; for example, if ebms act as conduits after several weeks of sustained antiangiogenic treatment, what are the roles of persistent vessels and their vasodilation response in relapse? How fast must ECs migrate and proliferate to rapidly repopulate ebms? Why have attempts to degrade ebms using inhibitors been ineffective in preventing rapid relapse^19^? In addition, numerous components of the basement membrane possess antiangiogenic properties, which may interfere with inhibiting relapse.

An alternative hypothesis is that partially regressed, persistent vessels, and not ebms, are the main drivers of the relapse of pathologic angiogenesis^18^. Persistent vessels have been shown to facilitate rapid relapse of corneal neovascularization upon return to a proangiogenic environment after four weeks of sustained vessel regression^18^. In the corneal model, ebms were not found to be reoccupied by ECs during relapse; instead, new capillary sprouts emerged alongside dormant ebms^18^. Rapid and aggressive relapse was instead shown to be linked to hyperdilation, hyperperfusion of the persisting vessels, and elevated inflammatory and angiogenic signaling during the relapse phase. The results differ with respect to ebms used as scaffolds during rapid relapse of pathology; however, these differences need to be interpreted with caution because MMPs, which are important for angiogenesis, are proteolytic enzymes that degrade BM into its derivatives, which possess antiangiogenic properties. MMPs also digest the ECM, releasing ECM-bound growth factors, which in turn attract inflammatory cells that express additional growth factors to drive angiogenesis. Degradation of the BM, as expected during the initial phase of angiogenesis, however, may result in the generation of BM-derived antiangiogenic fragments such as canstatin, tumstatin, and arrestin, whose antiangiogenic functions were discussed in detail above. These fragments may slow down or even inhibit angiogenesis to prevent relapse of the pathology. MMPs in relation to the BM, ECM and angiogenesis can therefore be considered “double-edged swords”; on the one hand they initiate angiogenesis through the proteolytic release of ECM-bound growth factors, and on the other hand, they inhibit angiogenesis via the generation of BM-derived anti-angiogenic fragments. It is likely that a fine balance is maintained to tightly regulate proangiogenic and antiangiogenic effects. Furthermore, observations that persistent corneal vessels (treatment-resistant vessels) may facilitate rapid relapse^18^ warrant further investigation to ascertain why these vessels do not fully regress in response to antiangiogenic treatment, such as anti-VEGF or dexamethasone treatment. Addressing this issue is important, as immunologic rejection of grafts following corneal transplantation and regrowth of choroidal neovessels in nAMD can occur via the reactivation of pre-existing pathologic vessels.

Regardless of whether ebms are reused, relapse after cessation of antiangiogenic treatment is an established fact. Where the reuse of ebms during relapse was not apparent, angiogenesis proceeded in the usual manner, with rapid vasodilation of persistent vessels followed by sprouting angiogenesis, albeit this process progressed away from the abandoned ebms. Angiogenesis relapses, regardless of the fate of ebms, are critical and warrant new strategies to inhibit relapse. In this context, observations that ebms are not reused as scaffolding by revascularizing EC is an important finding. In the study by Mukwaya et al., dense collagen IV deposits were found at the junction points between the embs and the persisting perfused vessels, which presumably completely seal off the ebms^18^ from the flow, thereby leading to irreversible EC apoptosis and the abandonment of vessel segments. Understanding this sealing process may lead to new treatment approaches by, for example, inducing excess collagen IV deposition in new sprouts to block new sprout growth and the connection of sprouts to parent perfused vessels. Rather than promoting rapid revascularization, collagen IV may be useful in the quest to inhibit pathologic angiogenic sprouting and relapse. This possibility leads to an interesting line of inquiry for future studies.

In summary, knowledge of the mechanisms of relapse of pathological angiogenesis is still in its infancy. Relapse should therefore be examined in greater detail at the phenotypic and regulatory levels, ideally in a disease- and tissue-specific manner. Models should be developed to recapitulate the conditions and timing of treatment in the clinical context, and the mechanism for relapse should eventually guide possible treatment strategies.
